# All records of rodents (Mammalia, Rodentia) and hares (Mammalia, Lagomorpha) in Georgia from 1855 through to 2022

**DOI:** 10.3897/BDJ.11.e108740

**Published:** 2023-08-23

**Authors:** Alexander Buknikashvili, Andrei Kandaurov, Giorgi Sheklashvili, Ioseb Natradze

**Affiliations:** 1 Institute of Zoology of Ilia State University, Tbilisi, Georgia Institute of Zoology of Ilia State University Tbilisi Georgia

**Keywords:** mammals, rodents, lagomorphs, dataset, distribution, Caucasus, species, occurrence.

## Abstract

**Background:**

In Georgia, there are 40 species from 21 genera and nine families of rodents, along with one species from a single genus and a family of hares. The dataset, which includes 3146 records, provides information regarding the presence of these species within 1559 locations. Of the total of 3146 records, 285 records from 281 locations belong to hares, while 2864 records from 1394 locations are associated with rodents. The dataset incorporates data sourced from various institutions, including the Collection of the Institute of Zoology of Ilia State University, the Georgian National Museum, the Collection of the Zoological Institute of Russian Academy of Sciences, the Zoological Museum of Moscow State University, as well as data extracted from literature and our own field data. The method for establishing the dataset included data compilation from all the above-mentioned sources. The dataset includes records from 1855 through to 2022.

**New information:**

In this publication, out of 3146 records, 14.9% are our new unpublished data. The unpublished data were collected from 2004 through to 2022. In total, 26% of all records are literature data, 50% are museum data and 24% are data collected by us. The dataset includes data obtained from literature sources, museum collections and the field observations. It includes occurrences of species found in the same locality, but within different years, resulting in separate records for each location. The dataset provided has the potential to significantly contribute to our understanding of the distribution of rodents and hares in Georgia.

## Introduction

In Georgia, the survey of rodents (Rodentia) was started in the 18^th^ century (Güldenstädt 1791, Pallas 1811) and was continued by Nordmann (1840). Further surveys were unsystematic, samples being collected for different European museums, including for Museums of the Russian Empire, as well as the Caucasus Museum (now the National Museum of Georgia). The samples stored in the 19^th^ century in the Caucasus Museum were published by Radde (1899).

The next step in the study of small mammals in Georgia was associated with the name of Konstantin Satunin, who worked in the Caucasus from 1893 to 1915. In his publications, Satunin (Satunin 1898, Satunin 1903, Satunin 1908, Satunin 1909, Satunin 1912, Satunin 1913, Satunin 1920) described a few new species, brought into order the taxonomy of Caucasian mammals and published the zoogeographic zoning for the Caucasus for the first time. It is noteworthy that K. Satunin is an author of the section on vertebrate animals in the publication of Radde (1899).

During the years 1916-1917, the Transcaucasian Experimental Station played a significant role in collecting samples of small mammals. The samples collected by the Station's employees are currently stored in the S. Janashia National Museum of Georgia. These samples were later used by many researchers and they have not lost their relevance even today.

Large-scale studies of small mammals are related to M.V. Shidlovskiy who was working at the Institute of Zoology and published more than 20 articles and monographs, including publications on rodents (Shidlovskiy 1919a, Shidlovskiy 1919b, Shidlovskiy 1938, Shidlovskiy 1940a, Anonymous 1940b, Shidlovskiy 1941a, Shidlovskiy 1941b, Shidlovskiy 1945, Shidlovskiy 1947a, Shidlovskiy 1947b, Shidlovskiy 1948, Shidlovskiy 1950, Shidlovskiy 1951, Shidlovskiy 1953, Shidlovskiy 1954a, Shidlovskiy 1954b, Shidlovskiy 1956, Shidlovskiy 1964, Shidlovsky 1976). The work "Rodents of Georgia - Faunistic structure and ecological-geographical spreading" (1948), existed as a report until 2013 and was published in 2013 (Shidlovskiy 2013). Parts of the work were published in articles dedicated to different regions of the country (Shidlovskiy 1948, Shidlovskiy 1950, Shidlovskiy 1951). The species and subspecies described by Shidlovskiy (1919a), Shidlovskiy (1919b) and Shidlovskiy (1938) and his ideas about the intraspecific heterogeneity of some species (Shidlovskiy 1954a, Shidlovskiy 1956) were not recognised by most of the zoologists at that time. However, these ideas are still actual and most of the taxa he described are considered valid species or subspecies today.

Valuable data about rodents and hare are in the publications of S. Kokhia (Kokhia 1950, Kokhia 1956, Kokhia 1958, Kokhia 1960, Kokhia 1961, Kokhia 1967, Kokhia 1968, Kokhia 1974a, Kokhia 1974b). These publications cover various aspects, including general rodentological information and specific studies dedicated to individual species. Information about the distribution of small mammals in Georgia is also given in the following publications (Papava (1940), Enukidze (1951a), Enukidze (1951b), Papava 1953, Papava 1955, Enukidze 1958, Papava 1959, Morgilevskaya 1960, Papava 1960, Morgilevskaya 1974, Enukidze et al. 1977, Enukidze and Zarqua 1985, Morgilevskaya 1989, Morgilevskaya and Tskipurishvili 1989). Furthermore, important information regarding the fauna of small mammals in certain administrative regions of Georgia is presented in the works of Avaliani (Avaliani 1969a, Avaliani 1969b, Avaliani 1970, Avaliani 1973, Avaliani 1976), As well as in the publications of parasitologists (Rodonaia 1956, Alania et al. 1964, Alania et al. 1971, Matsaberidze 1976, Kurashvili et al. 1977, Sagdieva and Kandaurov 1985, Kurashvili et al. 1989, Sagdieva et al. 2002).

An important role in the research of rodents in Georgia played S. Ognev (Ognev 1940, Ognev 1950), who described in detail the distribution, systematics and biology of rodents living here. Significant data about rodents of Georgia are in the publication Verestchagin (1959); however, these data are given on the maps, without naming toponyms and, therefore, they can be connected/linked to any place only approximately. In addition, data collected in Georgia are used in different publications of foreign researchers (Vorontsov et al. 1992, Lavrenchenko et al. 1994, Orth et al. 1996, Kotenkova 2002, Jaarola et al. 2004, Kryštufek and Vohralik 2005, Kryštufek and Vohralik 2009, Kotenkova et al. 2016).

Additionally, it, should be mentioned that, except for the Simon Janashia National Museum of Georgia, samples collected in Georgia during the research of rodents and hares, are stored in the collection of the Institute of Zoology of Ilia State University (Kandaurov and Tskhadaya 2014, Kandaurov et al. 2015, Tskhadaia et al. 2019).

The rodent distribution and systematics studies are underway (Kandaurov 1995, Bukhnikashvili 2004, Buknikashvili and Chkhikvadze 2004, Bukhnikashvili et al. 2012a, Bukhnikashvili et al. 2012b, Buknikashvili et al. 2013, Bukhnikashvili et al. 2015, Anonymous 2016, Yanchukov et al. 2020). Today, most of the territory of Georgia has been studied in terms of hare and the rodent species distribution, there being gaps left in high mountainous areas of the country. In high mountainous areas, above 2500 m, there are only 42 records from 27 locations and above 3000 m - only one record from one location.

## Sampling methods

### Study extent

The dataset includes information about 3146 records of 41 species of 22 genera of 10 families and two orders collected from 1855 through to 2022.

### Sampling description

The dataset includes data from literature collected from sources in Georgian, Russian and English languages, the museum data and data collected directly from field observations. Of the dataset, 76% is literature and collection data from 68 different sources. Of them, four are collections and 64 are publications. From these sources, we retrieved the maximum available data, such as date, habitat description, sampling place, closest settlement etc. We used the name of locations and, based on habitat descriptions mentioned in the publications or labels in the collection samples, we tried to identify coordinates using Google Earths services. A significant part of the records retrieved from literature sources is taken from those publications where the species distribution data first appeared for Georgian territory.

**Figure 1.**
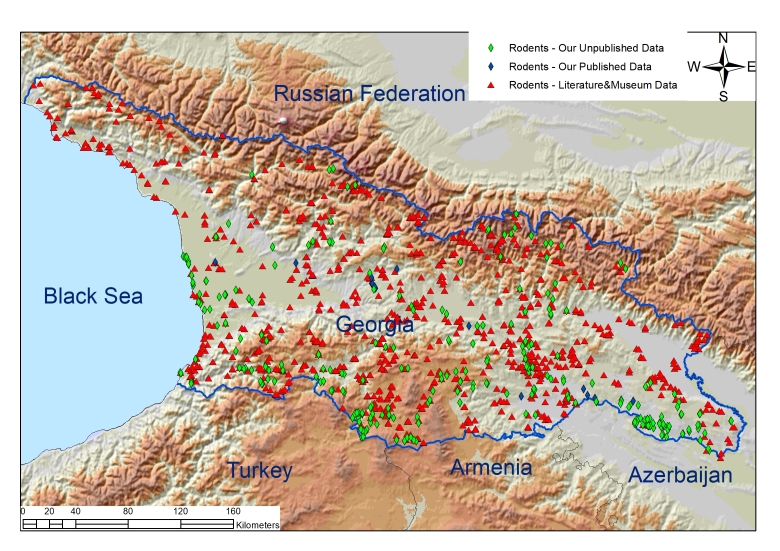
Map #1 Rodents records in Georgia.

**Figure 2.**
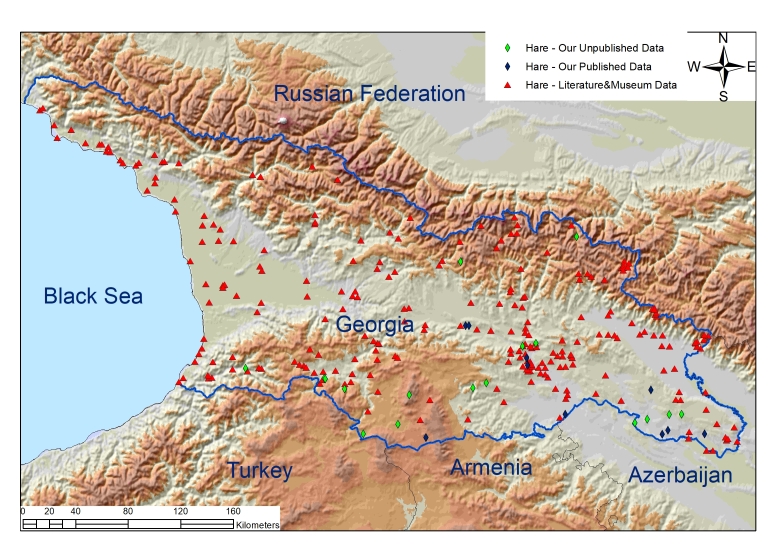
Map #2 Hares records in Georgia

There is a difference between the number of records obtained from literature and the number of records containing unpublished data on each species (Table 1, Fig. 1, Fig. 2).

Our field data were collected from 1982 through 2022. During surveys, most samples were caught using snap-traps (Hero traps), the lesser part trapped using live-traps and using pitfall traps (cylinders). Depending on the objectives, 20 to 50 traps were exposed with a distance between traps from five to ten metres (Sheftel 2018). In recent days, most animals have been caught using the Sherman live traps. Animal dissections and measurements are done following the recent protocols (Herbreteau et al. 2011). Only samples identified at the species level were included in the dataset.

The vole *Microtusrossiaemeridionalis* was recorded in Georgia in 2022 for the first time. That is a new species to the Georgian rodent fauna. The presence of this species was confirmed by molecular methods (Ratnasingham and Hebert 2007, Astrin and Stüben 2008, Astrin et al. 2016, Kumar et al. 2018, Sayers et al. 2021). The closest known occurrence of this species is in Turkey, where it lives in the mountains and on the Black Sea coast (Kryštufek and Vohralik 2005).

### Quality control

Records taken from literature and museums were included in the dataset only if we were able to determine their coordinates. For samples without coordinates obtained from old museum collections, published sources and our field data before 2004, we did georeferencing using Google Earth. The coordinates are given in degree decimal format in the WGS84 system. The precision of the coordinates depends on the source. In the case of our field observation, using the GPS device, it is presented in the dataset as having a precision of about 30-100 m. However, it should be mentioned that our original field data have a precision of four metres. In the case of data from literature and collections, we obtained an accuracy of about 800-1000 m. The spatial distribution of the finding points within the limits of the study area is shown on the map.

In addition, not all records in literature and the collection have dates. We were able to find information on dates for about 74.5% of records (Fig. 2).

## Geographic coverage

### Description

The dataset provides information about 3146 records of rodents and hares from 1559 locations in the country of Georgia (Fig. 1, Fig. 2). Georgia, covering an area of 69,700 km^2^, is located on the border of Europe and Asia. More than 80% of its territory is covered by ridges and plateaus. From the physical-geographical point of view, Georgia consists of five districts, namely: (i) Caucasus highland districts, (ii) Colchis District, (iii) Kura (Mtkvari) River Valley/lowland District, (iv) Lesser Caucasus District and (v) Volcanic Highland District of South Georgia (Ukleba 1981b).

Georgia's landscapes are also diverse, starting from the semi-deserts in Eastern Georgia and the humid subtropics in Colchis, ending with the snow glaciers in the Greater Caucasus chain. Georgia is a mainly mountainous country and, accordingly, the change in natural components according to the elevation is well expressed and, accordingly, there is a full spectrum of altitudinal zonation of landscapes (Ukleba 1981a).

Different researchers recognise a different number of landscape types in the Caucasus and particularly in Georgia (Beruchashvili 2000). We rely on Ukleba (1981b), according to whom there are 11 landscape types in Georgia: 1. Humid subtropical landscapes of Colchis lowland; 2. Thorn-bush steppe landscapes of dry subtropical plains; 3. Bluestem grass steppe landscapes of dry subtropical plains; 4. Steppes, shrublands and light forests landscapes of semi-arid subtropical plains and low-mountain; 5. Landscapes of the moderately humid subtropical plains; 6. Semi-desert landscapes of lowland plains; 7. Colchic forest landscapes of humid mountains; 8. Forest landscapes of the moderately humid eastern Transcaucasian mountains; 9. Landscapes of the mountain steppes; 10. High-mountain meadow landscapes (sub-alpine-alpine-meadow); 11. Nival landscapes and glaciers of the high mountains of the Greater Caucasus. A separate, azonal type is the floodplain landscape formed along the main rivers of Georgia, which consists of alder or willow in western Georgia and in the mountains and in the plains of eastern Georgia, so-called tugai-forests are formed and are characteristic only for the Caucasus and Central Asia.

Georgia is a part of the Caucasus Hotspot (Mittermeier et al. 2011), one of 36 biodiversity hotspots in the world, with high species and subspecies diversity.

Of 1559 locations, 479 locations which make up 30.7% are locations obtained during the surveys we conducted and, out of them, 333 (which make up 69.5%) are unpublished locations. Additionally, we increased the intensity studying of rodents and hares in the areas such as semi-arid and arid zones.

### Coordinates

40.94 N and 43.81 N Latitude; 39.66 E and 46.93 E Longitude.

## Temporal coverage

### Notes

**Figure 3.**
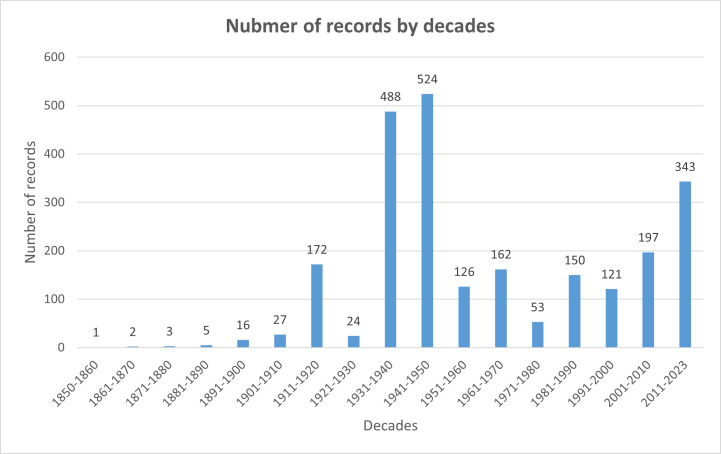
Number of records.

From 1855 through to 2022, surveys were carried out with different intensities. If we divide the survey period into decades, we will see that the most intensive research was conducted between 1931-1950 and from 2011 through 2022 (Fig. 3).

## Usage licence

### Usage licence

Creative Commons Public Domain Waiver (CC-Zero)

## Data resources

### Data package title

Rodent occurrence in Georgia

### Resource link

https://www.gbif.org/dataset/81394c45-1942-4689-bfc5-e053ed33b7cc

### Alternative identifiers

https://cloud.gbif.org/eca/resource?r=geo_rodents

### Number of data sets

1

### Data set 1.

#### Data set name

Rodent occurrence in Georgia

#### Data format

Darwin Core Archive (DwC-A)

#### Description

The dataset contains information on 3146 sampling points records for the 40 species of rodents and one species of hare in Georgia. The occurrences were recorded between the years 1855 and 2022 (Bukhnikashvili et al. 2023). Each record in the dataset contains the following information: species name, locality name, known event date, coordinates, coordinate uncertainty in metres, altitude, source of information and sources of georeference.

**Data set 1.** 

Column label	Column description
occurrenceID	Unique identifier of record.
kingdom	The full scientific name of the kingdom in which the taxon is classified.
phylum	The full scientific name of the phylum in which the taxon is classified.
class	The full scientific name of the class in which the taxon is classified
order	The full scientific name of the order in which the taxon is classified.
family	The full scientific name of the family in which the taxon is classified.
scientificName	Species full scientific (Latin) name including authorship and year.
locality	The specific description of the place of collection.
eventDate	Collection event date.
countryCode	Standard ISO 3166-1-alpha-2 country code.
decimalLatitude	The geographic latitude (in decimal degrees).
decimalLongitude	The geographic longitude (in decimal degrees).
geodeticDatum	Geographic coordinates reference system EPSG.
coordinateUncertaintyInMetres	Coordinate measurement accuracy (metres in case of GPS recordings, NA - if manually georeferenced). However, see the field "dataGeneralisations" for furher details.
minimumElevationInMetres	Minimum elevation above sea level.
maximumElevationInMetres	Maximum elevation above sea level.
associatedReferences	Source for the particular record.
georeferenceSources	The system used during the georeferencing.
basisOfRecord	The specific nature of the data record.
institutionCode	The code of the institution where data are stored.
collectionCode	The code of the collection.

## Additional information

Field data were collected under the permissions #2722/01; 2302/01; R/057-21, issued by the Ministry of Environmental Protections and Agriculture of Georgia.

## Figures and Tables

**Table 1.** Number of records for each species.

	Scientific name	Endemics and sub-endemics to the Caucasus Ecoregion (Zazanashvili et al. 2020)	Number of records	Literature & Museum data	Our published data	Our Unpublished data
	**Lagomorpha**					
	**Leporidae**					
	***Lepus***					
1	*Lepuseuropaeus* Pallas, 1778		285	258	9	18
	**Rodentia**					
	**Sciuridae**					
	***Sciurus***					
2	*Sciurusanomalus* Güldenstädt, 1785		71	69	0	2
3	*Sciurusvulgaris* Linnaeus, 1758		54	48	2	4
	**Hysteridae**					
	***Hystrix***					
4	*Hystrixindica* Kerr, 1792		5	0	4	1
	**Echimyidae**					
	***Myocastor***					
5	*Myocastorcoypus* (Molina, 1782)		42	17	7	18
	**Gliridae**					
	***Glis***					
6	*Glisglis* (Linnaeus, 1766)		117	107	5	5
	***Dryomys***					
7	*Dryomysnitedula* (Pallas, 1778)		110	88	15	7
	**Sminthidae**					
	***Sicista***					
8	*Sicistacaucasica* Vinogradov, 1925	+	3	3	0	0
9	*Sicistakluchorica* Sokolov, Kovalskaya & Baskevich, 1980	+	6	5	0	1
10	*Sicistakazbegica* Sokolov, Baskevich & Kovalskaya, 1986	+	8	7	0	1
	**Dipodidae**					
	***Scarturus***					
11	*Scarturuselater* (Lichtenstein, 1828)		6	3	0	3
12	*Scarturuswilliamsi* (Thomas, 1897)		2	0	2	0
	**Spalacidae**					
	***Nannospalax***					
13	*Nannospalaxxanthodon* (Nordmann, 1840)		41	22	4	15
	**Cricetidae**					
	***Cricetulus***					
14	*Cricetulusmigratorius* (Pallas, 1773)		77	67	7	3
	***Mesocricetus***					
15	*Mesocricetusbrandti* (Nehring, 1898)		97	55	20	22
16	*Mesocricetusraddei* (Nehring, 1894)	+	1	1	0	0
	***Prometheomys***					
17	*Prometheomysschaposchnikovi* Satunin, 1901	+	49	45	1	3
	***Myodes***					
18	*Myodesglareolus* (Thomas, 1906)		4	4	0	0
	***Ondatra***					
19	*Ondatrazibethicus* Link, 1795		8	0	3	5
	***Arvicola***					
20	*Arvicolaamphibius* (Linnaeus, 1758)		87	72	2	13
	***Chionomys***					
21	*Chionomysnivalis* (Martins, 1842)		30	26	0	4
22	*Chionomysgud* Satunin, 1909	+	70	60	2	8
23	*Chionomyslasistanius* Neuhäuser, 1936		9	9	0	1
24	*Chionomysroberti* (Thomas, 1906)	+	75	66	4	5
	***Microtus***					
25	*Microtusmajori* (Thomas, 1906)		188	151	21	26
26	*Microtusdaghestanicus* (Shidlovsky, 1919)	+	102	73	7	22
27	*Microtussocialis* (Pallas, 1773)		171	124	31	16
28	*Microtusobscurus* (Eversmann, 1841)		165	140	5	20
29	*Microtusrossiaemeridionalis* Ognev, 1924		1	0	0	1
	**Muridae**					
	***Meriones***					
30	*Merionestristrami* Thomas, 1892		8	7	0	1
31	*Merioneslibycus* Lichtenstein, 1823		66	21	3	42
	***Micromys***					
32	*Micromysminutus* (Pallas, 1771)		7	5	1	1
	***Apodemus***					
33	*Apodemusagrarius* Pallas, 1771		17	13	3	1
34	*Apodemusuralensis* Pallas, 1811		245	147	34	64
35	*Apodemuswitherbyi* (Thomas, 1902)		278	177	45	56
36	*Apodemusponticus* Sviridenko, 1936	+	74	47	19	8
37	*Apodemusmystacinus* Danford & Alston, 1877		54	39	1	14
	***Mus***					
38	*Musmusculus* Linnaeus, 1758		270	225	19	26
39	*Musmacedonicus* Petrov & Ruzic, 1983		55	15	18	22
	***Rattus***					
40	*Rattusrattus* (Linnaeus, 1758)		92	84	2	6
41	*Rattusnorvegicus* (Berkenhout, 1769)		96	90	1	5
